# Reaffirming the link between chronic phantom limb pain and maintained missing hand representation

**DOI:** 10.1016/j.cortex.2018.05.013

**Published:** 2018-09

**Authors:** Sanne Kikkert, Heidi Johansen-Berg, Irene Tracey, Tamar R. Makin

**Affiliations:** a Wellcome Centre for Integrative Neuroimaging, MRIB Centre, Nuffield Department of Clinical Neurosciences, University of Oxford, Oxford, United Kingdom; b Nuffield Division of Anaesthetics, University of Oxford, Oxford, United Kingdom; c Institute of Cognitive Neuroscience, University College London, London, United Kingdom

**Keywords:** Neuroimaging, Plasticity, Motor control, Amputees, Neuropathic pain

## Abstract

Phantom limb pain (PLP) is commonly considered to be a result of maladaptive brain plasticity. This model proposes that PLP is mainly caused by reorganisation in the primary somatosensory cortex, presumably characterised by functional degradation of the missing hand representation and remapping of other body part representations. In the current study, we replicate our previous results by showing that chronic PLP correlates with maintained representation of the missing hand in the primary sensorimotor missing hand cortex. We asked unilateral upper-limb amputees to move their phantom hand, lips or other body parts and measured the associated neural responses using functional magnetic resonance imaging (fMRI). We confirm that amputees suffering from worse chronic PLP have stronger activity in the primary sensorimotor missing hand cortex while performing phantom hand movements. We find no evidence of lip representation remapping into the missing hand territory, as assessed by measuring activity in the primary sensorimotor missing hand cortex during lip movements. We further show that the correlation between chronic PLP and maintained representation of the missing hand cannot be explained by the experience of chronic non-painful phantom sensations or compensatory usage of the residual arm or an artificial arm (prosthesis). Together, our results reaffirm a likely relationship between persistent peripheral inputs pertaining to the missing hand representation and chronic PLP. Our findings emphasise a need to further study the role of peripheral inputs from the residual nerves to better understand the mechanisms underlying chronic PLP.

## 1 Introduction

Following amputation, individuals often perceive painful sensations coming from their missing limb (Weeks, Anderson-Barnes, & Tsao, 2010). Phantom limb pain (PLP) is typically unresponsive to conventional analgesic therapies and poses a significant medical problem (Knotkova, Cruciani, Tronnier, & Rasche, 2012). The underlying cause of PLP remains poorly understood and extensively debated (Boström et al., 2014, Devor et al., 2014, Flor, 2002, Flor and Andoh, 2017, Flor et al., 2013, Flor et al., 2006, Foell and Andoh, 2014, Jutzeler and Curt, 2015, Moseley and Flor, 2012, Raffin et al., 2016, Makin et al., 2013, Makin et al., 2015).

An influential model attributes this neuropathic pain syndrome to ‘maladaptive plasticity’ (Flor et al., 2006, Flor et al., 1995, Lotze et al., 2001). Following arm amputation, the primary somatosensory missing hand cortex is deprived of a major source of sensory input. The maladaptive plasticity model suggests that this deprivation of inputs leads to cortical reorganisation, where the deprived cortex becomes responsive to inputs from its cortical neighbours. The displaced inputs from these cortical neighbours into the missing hand cortex are thought to trigger painful representations relating to the missing hand (i.e., PLP) and are therefore considered maladaptive. Support in favour of the maladaptive plasticity model stems from functional magnetic resonance imaging (fMRI) or magnetoencephalography (MEG) studies using tactile lips stimulation or lip movements in upper limb amputees with varying degrees of PLP. Amputees with worse PLP had greater cortical reorganisation, as estimated by measuring cortical distances between the lip and estimated missing hand representations (Birbaumer et al., 1997, Flor et al., 1998, Flor et al., 1995, Foell et al., 2014, Grüsser et al., 2001, Karl et al., 2001, Lotze et al., 2001) or activity levels in the missing hand cortex during lip movements (MacIver, Lloyd, Kelly, Roberts, & Nurmikko, 2008). Representations of the missing limb are less frequently investigated directly (Diers et al., 2010, Foell and Andoh, 2014, MacIver et al., 2008, Mercier et al., 2006, Raffin et al., 2016).

A more recent line of research highlighted peripheral PLP contributors, i.e., neuroma formation and ectopic firing in the residual nerves and/or dorsal root ganglia (Borghi et al., 2010, Vaso et al., 2014), emphasizing the potential for a maintained missing hand representation. While the maladaptive plasticity model has previously been associated with degradation of the missing hand representation and cortical reorganisation (Ramachandran & Hirstein, 1998), we recently reported that amputees with worse chronic PLP showed stronger activity in the primary sensorimotor missing hand cortex during phantom hand movements. We interpreted this result as evidence for more maintained representation (Makin, Scholz, et al., 2013). Our approach was based on empirical evidence demonstrating that phantom hand movements elicit central and peripheral sensorimotor signals different from those found during movement imagery (Raffin and Mattout, 2012, Reilly et al., 2006). By asking amputees to perform phantom hand movements, we directly targeted an otherwise latent phantom hand representation in the primary sensorimotor missing hand cortex (Kikkert et al., 2016). This is unlike previous studies using sensory lip stimulation, lip movements or imaginary phantom hand movements to determine reorganisation in the missing hand cortex (Birbaumer et al., 1997, Flor et al., 1998, Flor et al., 1995, Foell and Andoh, 2014, Grüsser et al., 2001, Karl et al., 2001, Lotze et al., 2001, MacIver et al., 2008).

The importance of replicating studies has previously been highlighted. For example, numerous studies have pointed at methodological confounds, such as inappropriate statistical power, which may compromise key findings in psychology and neuroimaging (Aarts et al., 2015, Button et al., 2013, Poldrack et al., 2017). Common to these critical papers is the agreement that while original studies offer tentative evidence, replications offer additional and confirmatory evidence. Given the impact of our original work on the pain community and the controversy it raised (Flor and Andoh, 2017, Flor et al., 2013, Jutzeler and Curt, 2015, Raffin et al., 2016, Vaso et al., 2014), we aimed to replicate our findings while closely following the methodology of our original study (Makin, Scholz, et al., 2013). We further extend the core findings of Makin, Scholz, et al. (2013) by addressing some previously raised criticisms (detailed further below).

## 2 Methods

### 2.1 Participants

The current data was collected as part of two larger studies detailed in https://osf.io/kd2yh/and https://osf.io/gmvua/, on which we previously published (Hahamy et al., 2017, van den Heiligenberg et al., 2018, van den Heiligenberg et al., 2017). Twenty-seven unilateral upper-limb amputees (mean age ± s.e.m. = 49.2 ± 2.4, ten right-arm amputees, see Table 1 for details) and thirty-one two-handed controls (age = 41.6 ± 2.4, eleven left-hand dominant controls) were recruited. Eleven of the twenty-seven amputees participating in the current study also participated in our original study (i.e., A17–A27, see Table 1; Makin, Scholz, et al., 2013). We note, however, that novel data was acquired for each participant: the eleven participants that overlapped across studies were scanned again for the purpose of the current study.

**Table 1:** **Demographic and clinical details.** N = no; Y = yes; Amp. = amputation; Amp. level: 1 = through shoulder, 2 = above elbow, 3 = through elbow, 4 = below elbow, 5 = wrist and below; Side = side of amputation; dominant = hand dominance prior to amputation (based on self-report); A = ambidextrous; L = left; R = right; PLS = phantom limb sensation; PLP = phantom limb pain; Inf. = infection; Pros. usage = prosthetics usage: 0 = never, 1 = rarely, 2 = occasionally, 3 = daily -less than 4 hours, 4 = daily - more than 4 hours, 5 = daily -over 8 hours; Treatment = pharmacological treatment for PLP.

	Tested in Makin, Scholz, et al. (2013)	Scanner	Age	Age at amp.	Amp. level	Side/dominant	PLS intensity	PLS frequency	Chronic PLS	PLP intensity	PLP frequency	Chronic PLP	Transient PLP	Chronic residual arm (stump) pain	Cause of amp.	Pros. usage	Treatment	Telescoping
A1	N	Prisma	71	53	2	R/A	20	1	20	85	2	42.5	20	0	Trauma	5	N	Y
A2	N	Prisma	46	26	2	R/R	70	1	70	90	1	90	50	0	Trauma	5	N	Y
A3	N	Prisma	64	31	2	L/R	100	1	100	40	5	8	10	0	Trauma	0	N	Y
A4	N	Prisma	58	54	2	L/R	90	1	90	0	0	0	0	0	Inf.	0	N	Y
A5	N	Prisma	57	26	2	R/R	80	1	80	90	5	18	40	0	Trauma	1	N	Y
A6	N	Prisma	50	47	4	L/R	0	0	0	0	0	0	0	20	Trauma	4	N	N
A7	N	Prisma	52	23	2	R/R	100	1	100	80	2	40	50	15	Trauma	0	N	N
A8	N	Prisma	68	40	4	R/R	80	5	16	0	0	0	0	12.5	Trauma	4	N	N
A9	N	Prisma	40	28	4	R/L	70	2	35	40	2	20	30	70	Trauma	0	N	Y
A10	N	Prisma	47	30	4	L/R	80	1	80	30	1	30	30	8	Trauma	5	N	Y
A11	N	Verio	41	27	2	R/L	100	1	100	80	1	80	60	100	Trauma	2	N	N
A12	N	Verio	48	17	2	L/R	100	1	100	75	3	25	0	10	Trauma	2	N	N
A13	N	Verio	37	27	2	L/R	90	1	90	40	3	13.3	10	16.7	Trauma	2	N	Y
A14	N	Verio	64	33	4	R/R	75	1	75	0	0	0	0	0	Trauma	5	N	Y
A15	N	Verio	29	24	1	L/R	80	2	40	70	4	17.5	0	0	Trauma	2	N	Y
A16	N	Verio	32	31	2	L/R	100	1	100	100	2	50	10	0	Trauma	2	Y	N
A17	Y	Prisma	59	44	4	L/R	10	5	2	0	0	0	0	0	Trauma	5	N	N
A18	Y	Verio	57	20	4	L/L	90	4	22.5	60	1	60	60	0	Trauma	5	N	N
A19	Y	Verio	59	40	2	L/L	96	2	48	91	4	22.75	0	47.5	Trauma	0	Y	N
A20	Y	Verio	58	27	2	L/R	70	2	35	90	2	45	20	15	Inf.	5	N	Y
A21	Y	Verio	53	28	4	L/R	20	2	10	40	2	20	0	15	Trauma	5	N	Y
A22	Y	Verio	46	38	4	L/R	90	1	90	94	1	94	90	20	Trauma	0	Y	N
A23	Y	Verio	24	18	4	R/L	100	1	100	90	2	45	0	35	Trauma	0	N	Y
A24	Y	Verio	49	37	2	L/R	20	1	20	80	3	26.7	0	0	Trauma	1	Y	N
A25	Y	Verio	50	45	2	L/L	90	1	90	70	1	70	40	16.7	Tumour	2	N	N
A26	Y	Verio	25	18	5	L/R	100	1	100	30	1	30	10	18	Trauma	2	N	N
A27	Y	Verio	45	20	4	R/L	50	1	50	50	5	10	0	40	Trauma	2	N	Y

Ethical approval was granted by Oxford University's Medical Sciences committee (Ref: MSD-IDREC-C2-2014-003 and MS-IDREC-C2-2015-012). Written informed consent was obtained from all participants prior to the study. To compare between groups, amputees' phantom/intact hand and residual arm was matched to controls' non-dominant/dominant hand and non-dominant arm, respectively (note that the proportion of right-arm amputees and left-hand dominant controls was similar: 37% and 35.5% respectively; X^2^_(1)_ = .02, *p* = .90).

### 2.2 Pain ratings

Amputees rated the frequency of PLP, as experienced within the last year, as well as the intensity of worst PLP experienced during the last week (or in a typical week involving PLP; see Table 1). Chronic PLP was calculated by dividing worst PLP intensity (scale 0–100: ranging from no pain to worst pain imaginable) by PLP frequency (1 = all the time, 2 = daily, 3 = weekly, 4 = several times per month, and 5 = once or less per month). This approach reflects the chronic aspect of the PLP as it combines both frequency and intensity (Draganski et al., 2006, Kikkert et al., 2016, Kikkert et al., 2017, Lyu et al., 2016, Makin et al., 2013, Makin et al., 2015). We have previously shown excellent inter-study consistency for this measure of PLP chronicity (Kikkert et al., 2017). Similar measures were obtained for non-painful phantom sensation vividness and residual limb (stump) pain. A transient PLP intensity rating (scale 0–100, as above) was also obtained prior to scanning. PLP properties were further quantified using an adapted version of the McGill Pain Questionnaire (Melzack, 1987). For each given quantitative PLP description, amputees provided an intensity rating (scale 0–100, as above). Table 2 represents the percentage of amputees that rated a certain qualitative PLP experience > 0. We did not identify a significant difference in chronic PLP severity between amputees experiencing telescoping and amputees not experiencing telescoping (*U* = 66.4, *p* = .23).

**Table 2:** **Qualitative PLP experiences.** % of amputees = percentage of amputees rating a given qualitative PLP description as greater than 0 (i.e., indicating this description matched their PLP experience). PLP = phantom limb pain.

PLP Sensation description	% of amputees
**Mechanical**	
Pulsing	40.7
Stabbing	63.0
Cutting	14.8
Pushing	14.8
Pinching	7.4
Squashing	29.6
**Temperature**	
Hot	18.5
Burning	40.7
Chilly	14.8
Freezing	11.1
**Other**	
Pricking	22.2
Tingling	59.3
Itchy	37.0
Electric current	51.9
Shooting	51.9
Exploding	3.7

### 2.3 Scanning procedures

Participants were visually instructed to move their intact hand (all fingers flexion/extension), phantom hand (as the intact hand), residual arm (elbow flexion), lips (blowing kisses) and feet (bilateral toes) in a block-design fashion, as well as their intact arm (not reported here). Each of the six movement conditions was repeated four times in a semi-counterbalanced protocol, alternating 12 s of movement with 12 s of rest. Movement pace was instructed at 0.5 Hz. Importantly, volitional phantom hand movements are distinguishable from imagined movements, as is supported by empirical evidence demonstrating that phantom limb movements elicit both central and peripheral motor signals that are different from those found during movement imagery (Kikkert et al., 2017, Raffin et al., 2012, Raffin et al., 2012, Reilly et al., 2006). We therefore clarified with each participant that they should make actual rather than imagined phantom hand movements (Raffin et al., 2012, Reilly et al., 2006). By asking amputees to perform phantom hand movements, we directly targeted otherwise latent phantom hand representation in the primary sensorimotor missing hand cortex (Kikkert et al., 2016). The experimenter explained the tasks to be performed inside the scanner and participants were given extensive training on the expected movements. As PLP is associated with phantom hand movements and phantom hand motor control (Kikkert et al., 2017), emphasis was given on making simple movements. If participants were unable to perform full phantom hand movements, they were asked to attempt to perform the movements to their best ability.

### 2.4 MRI data acquisition

MRI images were collected using either a 3 tesla Siemens Verio MRI scanner or a 3 tesla Siemens Prisma MRI scanner, with a 32-channel head coil (see Table 1). MRI data acquisition, preprocessing and analysis followed standard procedures. A high-resolution T1-weighted sequence was used to acquire a structural image (Verio: TR = 2040 ms, TE = 4.7 ms, flip angle = 8°, voxel size = 1 mm^3^; Prisma: TR = 1900 ms, TE = 3.97 ms, flip angle = 8°, voxel size = 1 mm^3^). Functional images based on the blood oxygenation level-dependent (BOLD) signal were obtained using a multiple gradient echo-planar T2*-weighted pulse sequence on the Verio MRI scanner (TR = 2000 ms, TE = 30 ms, flip angle = 90°, voxel size = 3 mm^3^, 46 axial slices, volumes = 300) and a multiband T2*-weighted pulse sequence on the Prisma MRI scanner (TR = 1500 ms, TE = 32.40 ms, flip angle = 75°, voxel size = 2 mm^3^, 72 transversal slices, volumes = 400, between-slice acceleration factor = 4). Coverage included the whole cortex, and partially the cerebellum. Additional dummy volumes were acquired before the scan. Field maps were acquired for field unwarping.

### 2.5 MRI data analysis

**Fig. 1:**
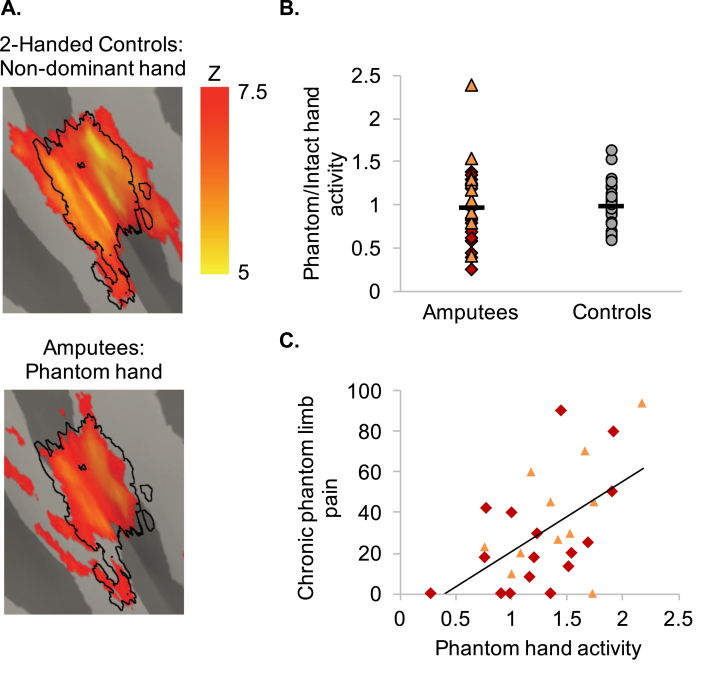
**Chronic phantom limb pain relates to maintained cortical phantom hand representation.** (A) Activity for controls (top) and amputees (bottom) during non-dominant/phantom hand versus feet movements (respectively). Black outlines define the boundaries of the ‘missing hand ROI_1_’. (B) Activity in the primary sensorimotor missing hand cortex (ROI_1_) during phantom hand movements did not significantly differ from activity during non-dominant hand movements in two-handed controls (top; see also Figure A.1 for a lack of a significant between-group difference in and around the the primary sensorimotor missing hand cortex in a whole-brain analysis). Horizontal lines represent the group averages. Red diamonds represent the sixteen amputees in the strict replication sample [i.e., the amputees that were not tested in Makin, Scholz, et al. (2013)]. The orange triangles represent the eleven amputees that also participated in Makin, Scholz, et al. (2013). Grey circles represent the two-handed control participants. (C) Amputees suffering worse chronic phantom limb pain activated the missing hand cortex more strongly during phantom hand movements (bottom).

All imaging data were processed using FSL version 5.0 (https://fsl.fmrib.ox.ac.uk/fsl/fslwiki; Smith et al., 2004, Woolrich et al., 2009). Cortical surface reconstructions, used for visualisation of the fMRI results, were produced using Workbench (http://www.humanconnectome.org). To align the missing/non-dominant hand hemisphere, data of individuals with a missing/non-dominant right hand was flipped on the mid–sagittal plane prior to all analysis (Bogdanov et al., 2012, Diers et al., 2010, Foell and Andoh, 2014, Lotze et al., 2001, Raffin et al., 2012). To ensure that this procedure did not impact our findings, we repeated the main correlational analysis described in the results (Fig. 1C) using a brain flipping regressor and found no differences in results (see Appendix A: Supplementary materials).

Common pre-processing steps for fMRI data were applied to each individual run, using FSL's Expert Analysis Tool FEAT (version 6.00): motion correction using FMRIB's Linear Image Registration Tool [MCFLIRT; Jenkinson, Bannister, Brady, & Smith (2002)], brain extraction using automated brain extraction tool BET (Smith, 2002), spatial smoothing using a 5 mm full width at half maximum Gaussian kernel, and high pass temporal filtering with a 150 s cut-off for the scans acquired using the Verio scanner and 185 s for the scans acquired using the Prisma scanner.

Functional data were aligned to structural images initially using FMRIB's Linear Image Registration Tool [FLIRT; Jenkinson and Smith, 2001, Jenkinson et al., 2002] and optimised using boundary based registration (Greve & Fischl, 2009). Structural images were transformed to MNI standard space using nonlinear registration tool FNIRT, and the resulting warp fields were applied to the functional statistical images.

First-level parameter estimates were computed using a voxel-based general linear model (GLM) based on the double-gamma hemodynamic response function and its temporal derivatives. Time series statistical analysis was carried out using FILM (FMRIB's Improved Linear Model) with local autocorrelation correction. Estimated head motion parameters (estimated by MCFLIRT) were added to the model to remove residual motion effects. Contrasts were defined for phantom/non-dominant hand movements versus rest, residual arm movements versus rest, and lip movements versus rest. A further contrast was defined to aid region of interest (ROI) selection: phantom/non-dominant hand versus feet movements (see below for details). This latter contrast ensured that we only selected body-part specific activation for ROI creation.

Group-level analysis was performed using FMRIB's Local Analysis of Mixed Effects (Woolrich, Behrens, Beckmann, Jenkinson, & Smith, 2004). Whole-brain group differences were tested for phantom/non-dominant hand movements versus rest and lip movements versus rest (see Figure A.1A and Figure A.1B, respectively). A regressor of no interest was included in the model to account for differences across the two studies' imaging protocols. Z-statistic images were thresholded using clusters determined by Z > 3.1 and *p* < .05 family-wise-error-corrected cluster significance thresholding was applied.

Hand ROIs were created based on the non-thresholded and uncorrected top 400 voxels activated during hand versus feet movements in a group-level analyses, surrounding the contralateral hand knob. The main ROI (ROI_1_) was created based on averaged phantom/non-dominant hand movements across the amputee and control groups [as in our original study; Makin, Scholz, et al., (2013)]. Averaged percent signal change was extracted from each participant's first-level analysis for the phantom/non-dominant hand (or other body part) movements versus rest, though note that similar results were observed when using a phantom/non-dominant hand versus feet movements contrast. Group differences were calculated by dividing individual values from the ‘missing hand’ ROI by the corresponding values from the ‘intact hand’ ROI. Correlations were investigated using values extracted from the missing hand ROI. To ensure that differences across the two studies' imaging protocols did not impact our findings, we repeated our main analysis using a regressor for scanner type and found no difference in results (see Appendix A: Supplementary materials).

It has been suggested that our original analysis misestimated the ‘true’ missing hand area, as our original ROI was based on conjunct phantom/non-dominant hand movement activity in amputees and controls (Flor and Andoh, 2017, Flor et al., 2013). We therefore constructed two additional primary sensorimotor missing hand cortex ROIs: ROI_2_ was created based on phantom hand movements in the amputee group only [although we note that this approach has been highlighted to result in statistical biases; Kriegeskorte, Simmons, Bellgowan, & Baker (2009)] and ROI_3_ was created based on controls' non-dominant hand movements. These ROIs were used to confirm our key findings, as detailed in the results section and Appendix A: Supplementary materials. ROI_3_ was further used to assess test-retest reliability of fMRI activity levels across our original Makin, Scholz, et al. (2013) and the current study.

### 2.6 Statistical analysis

Statistical analysis was performed using SPSS (version 21) and Matlab (version 9.1). When assessing group differences by means of independent-samples *t*-tests we tested for homogeneity of variances using Levene's test for homogeneity of variances. If this assumption was violated, equal variances was not assumed and data is reported accordingly. Data were inspected for normality violations using the Shapiro–Wilk test. As the chronic PLP ratings were not normally distributed we employed a Box–Cox transformation to improve the distribution of the data: A constant of .1 was added to the chronic PLP ratings and a Box–Cox transformation with a lambda of .42 was applied. Non-parametric tests were utilised when needed. Standard approaches were used for statistical analysis, as mentioned in the Results section.

## 3 Results

We first aimed to replicate the main results of Makin, Scholz, et al. (2013) by only examining participants that were not tested in our original experiment. As reported previously, we did not find a significant group difference in primary sensorimotor missing hand cortex activity during phantom/non-dominant hand movements (ROI_1_: t_(23.6)_ = -1.51, *p* = .15). Next we repeated our previous study's main analysis (Makin, Scholz, et al., 2013), demonstrating a positive correlation between chronic PLP and activity in the primary sensorimotor missing hand cortex during phantom hand movements. As found before, amputees with worse chronic PLP showed greater activity in the missing hand cortex during phantom hand movements (ROI_1_: r = .54, *p* = .03).

We further extended the results of Makin, Scholz, et al. (2013) by pooling across the replication sample and the amputees that also participated in the previous experiment (though note that novel data was acquired for the purpose of the present analysis). We first examined which areas amputees recruited during phantom hand movement compared to controls' non-dominant hand. Amputees showed increased recruitment of bilateral insula (Figure A.1A; Table A.1), but not of sensorimotor cortex. This latter observation was confirmed using ROI analysis, showing no significant difference in primary sensorimotor missing hand cortex activation between the amputee and control groups (Fig. 1B; ROI_1_: t_(45.6)_ = -.81, *p* = .42). Amputees' phantom hand movement activity levels in the primary sensorimotor missing hand cortex did not significantly correlate with amputees' level of amputation (ROI_1_: r_s_ = .09, *p* = .64). As observed in our original study (Makin, Scholz, et al., 2013) and the replication analysis above, a significant correlation was found between phantom hand movement activity in the missing hand cortex and chronic PLP across the twenty-seven amputees (Fig. 1C; ROI_1_: r = .49, *p* < .01), that was not driven by experience of non-painful phantom sensations (partial correlation: ROI_1_: r_s_ = .45, *p* = .02).

To confirm that our results were not dependent on ROI selection criteria (Flor and Andoh, 2017, Flor et al., 2013), we constructed an additional ROI based on phantom hand movement activity in the amputee group only (ROI_2_; see Methods section for further details). Using this ROI_2_, we again found a significant correlation between chronic PLP and primary sensorimotor missing hand cortex activity during phantom hand movements (ROI_2_: r = .53, *p* < .01), regardless of non-painful phantom sensations (partial correlation: ROI_2_: r_s_ = .46, *p* = .02).

It was also suggested that activity in the missing hand cortex could be facilitated by an extended or shifted residual arm representation (Flor and Andoh, 2017, Flor et al., 2013), which was recently associated with PLP (Raffin et al., 2016). However, amputees did not show more activity in the primary sensorimotor missing hand cortex during residual arm movements compared to controls (ROI_1_: t_(56)_ = −.62, *p* = .54). There was a trend towards lower activity in amputees' missing hand cortex during residual arm movements compared to controls when only considering amputees with below-elbow amputations (*U* = 121, *p* = .08; see Makin, Cramer, et al. (2013) for similar results). Moreover, amputees' activity in the primary sensorimotor missing hand cortex was significantly higher during phantom hand movements compared to residual arm movements (ROI_1_: t_(26)_ = 7.20, *p* < .01). No significant relationship existed between chronic PLP and residual arm activity (ROI_1_: r = .28, *p* = .16). Furthermore, the observed correlation between chronic PLP and strength of missing hand cortex activity during phantom hand movements was independent of residual arm/prosthesis usage [partial correlation, regressing out residual arm/prosthesis usage; ROI_1_: r = .49, *p* = .01; as measured using questionnaires described and validated in Hahamy et al. (2017) and Makin, Cramer, et al. (2013)].

Next, we revisited the potential relationship between chronic PLP and lip representation remapping into the primary sensorimotor missing hand cortex, as such remapping has been considered an indicator of maladaptive plasticity (Flor et al., 1995, Flor et al., 1998, Flor et al., 2006). As reported previously (Makin et al., 2013, Makin et al., 2015), we could not find a significant group difference in activity in the missing hand ROI during lip movements (ROI_1_: *U* = 377, *p* = .52). Furthermore, no significant correlation was found between chronic PLP and missing hand cortex activity during lip movements (ROI_1_: r_s_ = −.31, *p* = .11). We found a significant difference between phantom hand and lip movement activity correlations with chronic PLP (ROI_1_: z = 3.08, *p* < .01), demonstrating that in the current dataset, as in the original publication, the maintained representation model was statistically better supported than the maladaptive plasticity model.

Finally, we assessed test-retest reliability across the original (Makin, Scholz, et al., 2013) and current study's main fMRI measures using interclass correlations (two-way random-model, consistency type) for the eleven individuals that participated in both studies. ICC values range from 0 to 1: ICC values < .4 are considered poor, .4 to .59 fair, .6 to .74 good, and >.75 suggest excellent inter-study consistency (Fleiss, Levin, & Cho Paik, 2003). We found excellent inter-study consistency for phantom hand movement activity levels and poor inter-study consistency for lip and residual arm movement activity levels in the primary sensorimotor missing hand cortex (see Table 3).

**Table 3:** **Test-retest reliability of fMRI activity levels in the missing hand ROI.** Averaged percent signal change was extracted from each participant's first-level analysis using ROI_3_ (defined by control participants' non-dominant hand movement activity). To ensure our ICC values were minimally affected by differences in scanner type, we normalised activity levels by dividing by intact hand movement activity levels extracted from an intact hand ROI. ICC = intraclass correlations; CI = 95% confidence interval.

	ICC-Value	ICC-CI
Phantom/intact hand movement activity	.75	.07 – .93
Lip/intact hand movement activity	−.22	−3.54 – .67
Residual arm/intact hand movement activity	.15	−2.14 – .77

## 4 Discussion

Here we confirm that chronic PLP associates with stronger activity in the primary sensorimotor missing hand cortex during phantom hand movements. This was observed regardless of ROI selection, demonstrating the robustness of our findings. Phantom hand movement activity was distinct from residual limb movements activity and the relationship between chronic PLP and missing hand cortex activity during phantom hand movements was not related to the residual arm representation. We further confirm our previous observation that amputees recruit bilateral insula more during phantom hand movements compared to when controls make non-dominant hand movements. The primary sensorimotor missing hand cortex and insula both receive afferent information originating from the injured primary afferent nerve. As such, ectopic firing caused by assault to the residual nerves and/or dorsal root ganglia following amputation should result in aberrant inputs into both cortical terminals, potentially explaining the observations described in this study. Collectively, our findings are compatible with recent evidence highlighting the role of aberrant peripheral inputs in driving PLP (Vaso et al., 2014).

Maladaptive reorganisation is still widely assumed to play a key role in PLP, as well as a range of other chronic pain conditions, with important implications for designing clinical treatments. Indeed, over the years the maladaptive plasticity model has been expanded in an attempt to explain other neuropathic pain syndromes, such as complex regional pain syndrome (Moseley & Flor, 2012), painful trigeminal neuropathy (Gustin et al., 2012) and neuropathic pain following spinal cord injury (Wrigley, Press, et al., 2009). While some treatments based on the maladaptive plasticity model have shown effective in relieving PLP (Chan et al., 2007, Ortiz-Catalan et al., 2016), placebo controls are often missing and the underlying mechanisms for such pain relief remain under discussion (Jutzeler and Curt, 2015, Mezue and Makin, 2016, Thieme et al., 2016). A key assumption of the maladaptive plasticity model is that cortical reorganisation is triggered by input loss (e.g., through arm amputation), thereby promoting remapping of adjacent representations (e.g., of the lips) into the deprived cortex, leading to a mismatch of body-part inputs. However, despite arm amputation, a detailed preserved missing hand representation can be observed in the primary sensorimotor missing hand cortex when amputees make phantom hand movements (Bruurmijn et al., 2017, Kikkert et al., 2016, Makin et al., 2013, Makin and Bensmaia, 2017, Mercier et al., 2006, Raffin et al., 2012). Furthermore, displaced signals in the primary somatosensory cortex (e.g., evoked by brain stimulation) do not trigger pain sensations (Flesher et al., 2016, Mazzola et al., 2012). Lastly, while local lip representation shifts occur following arm amputation, these do not invade the missing hand territory (Makin et al., 2015, Raffin et al., 2016).

It is conceptually and empirically possible that persistent representation can spatially coincide with reorganisation (Kikkert et al., 2016). However, both in previous studies (Makin et al., 2013, Makin et al., 2015) and here, we could not identify lip overrepresentation in the missing hand territory nor a relationship between displaced body-parts representations and chronic PLP. It is important to note a few methodological differences between the studies supporting and rebutting the invasion of lip representation into the missing hand territory. First, in our original and present studies reorganisation was assessed based on average activity levels. Conversely, in the original series of studies founding the maladaptive plasticity model (Birbaumer et al., 1997, Flor et al., 1998, Flor et al., 1995, Foell et al., 2014, Grüsser et al., 2001, Karl et al., 2001, Lotze et al., 2001), reorganisation was measured based on (Euclidian) distances from the facial activity's center of gravity (or peak in activity) to an estimated missing hand representation. It has been argued that this latter approach is more sensitive to subtle changes in the boundaries of the face representation, which might be averaged out using the former approach. However, we previously demonstrated that the cortical shifts apparent with centre of gravity distances analysis are localised, and do not convey remapping of facial inputs into the missing hand territory (Makin et al., 2015; see Raffin et al. (2016) for similar results). Second, in our studies we used an active motor task to probe lip representation, whereas the original studies used passive tactile face stimulation to demonstrate maladaptive reorganisation. However, we do not think that this methodological difference is underlying the inconsistent results. Movement-induced lip activity in the primary sensorimotor cortex was previously used to demonstrate lip remapping in amputees (Lotze et al., 2001, MacIver et al., 2008) and passive facial stimulation has recently been reported to not show reorganisation (Philip, Valyear, Cirstea, & Frey, 2017). Finally, our measure of chronic PLP differs from other studies concerning the neural basis of PLP, who used e.g., the pain intensity scale of the West Haven–Yale Multidimensional Pain Inventory (Flor et al., 1995) or a visual-to-analog rating of chronic PLP intensity experienced in the past 3 months (Raffin et al., 2016). Our PLP measure combines both intensity and frequency of PLP episodes, and thus aims to capture the chronicity of this condition [see also Draganski et al. (2006) and Lyu et al. (2016)]. Importantly, this measure was demonstrated to correlate with motor control of the phantom hand and was found to be highly consistent across studies (Kikkert et al., 2017). When comparing the correlation coefficients of chronic PLP with hand and lip movement activity in the missing hand cortex, we observed that activity during phantom hand movements was a better predictor of chronic PLP variability in the current dataset. This suggests that maintained missing hand representation is a more appropriate neural correlate of chronic PLP than maladaptive reorganisation. Indeed, evidence is growing to support a maintained representation model of chronic PLP (Blume et al., 2014, Borghi et al., 2010, Vaso et al., 2014, Yanagisawa et al., 2016), emphasising a need to investigate the role of maintained inputs (and/or organisation). Further studies are needed to address these differences in results across studies.

As proposed in early theories (Nystrom & Hagbarth, 1981) and emphasized again recently (Vaso et al., 2014), spontaneous activity from residual damaged nerves can prevail after amputation and, as such, carry information relating to the missing hand to the brain. This likely input seems to powerfully contribute to maintained representation that becomes unmasked during phantom hand movements. If this is correct, then amputees with worse chronic PLP (more residual peripheral inputs) will show stronger activity in the missing hand cortex during phantom hand movements, as observed in the previous (Makin, Scholz, et al., 2013) and current study [see also Kikkert et al. (2017)]. Reducing peripheral disturbances would then consequently reduce both PLP and maintained activity in the primary sensorimotor cortex. However, while local anaesthesia block has been reported to reduce PLP, it does not eliminate PLP in all cases (Birbaumer et al., 1997, Borghi et al., 2010, Flor, 2002, Flor et al., 2006, Nystrom and Hagbarth, 1981). A potential reason for the poor clinical efficacy of peripheral nerve block in PLP treatment, is that a full blocking of C-fibers is difficult to achieve (Serra et al., 2015). A further explanation that has been proposed is that much of the spontaneous (background) ectopic discharge originates from the dorsal root ganglia. Dorsal root ganglia electrogenesis could account for the therapeutic failure of neuroma, nerve, and plexus infiltration because these distal blocks do not affect the dorsal root ganglia ectopia. Indeed, Vaso et al. (2014) demonstrated that locally anaesthetising the dorsal root ganglia, thereby preventing ectopic signals from the dorsal root ganglia to reach the central nervous system, consistently attenuated and often completely eliminated PLP as well as non-painful phantom limb sensations in amputees. The maintenance of nociceptive peripheral signals following amputation may also be a potential source for the previously observed association between PLP and deteriorated motor control (Gagné et al., 2009, Kikkert et al., 2017): It is possible that aberrant inputs from the residual nerves to the primary sensorimotor missing hand cortex disrupts the functioning of the sensorimotor system, leading to deteriorated phantom hand motor control. We tentatively suggest that primary sensorimotor cortex activity may not causally drive PLP, but instead could be a secondary consequence of peripheral disturbances. Further work is needed to better dissect the contributory factors.

## Conflict of interest

The authors declare no conflict of interest.
