# Supplementary material for: Reaffirming the link between chronic phantom limb pain and maintained missing hand representation
Source: Cortex. 2018 Sep;106:174–84. doi: 10.1016/j.cortex.2018.05.013 (PMC6143485; doi:10.1016/j.cortex.2018.05.013)
Supplement: Multimedia component 1 [file mmc1.docx]

**Supplementary Figures**

**Figure A.1: Whole brain group comparisons.** (A) Whole-brain group comparison of phantom/non-dominant hand movements in amputees/controls, respectively. Brain laterality was aligned with respect to the missing hand, such that for amputees missing their right hand (n=10) or control participants that were left-hand dominant (n=11) the brain was flipped on the mid-sagittal axis prior to group analysis. Amputees show increased excitability in bilateral insula, anterior supramarginal gyrus, and prefrontal cortex, as well as of the pallidum and anterior cingulate cortex of the missing hand hemisphere (see Table A.1 for a detailed overview of peak activity) during phantom hand movements, compared to two-handed controls. (B) Whole-brain group comparison during lip movements in amputees and controls. There were no significant differences in activation. As above, brain laterality was aligned with respect to the missing and non-dominant hand for amputees and controls respectively. A=anterior; P=posterior. White arrows indicate the central sulcus.

|  | Hemisphere | Z-value | x | y | z |
| --- | --- | --- | --- | --- | --- |
| Insular Cortex | Missing | 5.1 | 44 | 4 | -2 |
|  | Intact | 4.8 | -52 | 12 | 0 |
| Prefrontal cortex | Missing | 4.3 | 48 | 42 | 0 |
|  | Intact | 4.2 | -38 | 36 | 12 |
| Supramarginal gyrus | Missing | 4.6 | 60 | -30 | 40 |
|  | Intact | 4.1 | -56 | -36 | 42 |
| Pallidum | Missing | 4 | 16 | -2 | -2 |
| Anterior cingulate cortex | Missing | 3.9 | 4 | 16 | 28 |

**Table A.1: Cluster peak activity parameters for whole-brain contrasts between amputees and controls.** Missing hemisphere refers to the hemisphere contralateral to the missing/non-dominant hand. Intact hemisphere refers to the hemisphere contralateral to the intact/dominant hand. X- y- and z coordinates are in MNI space.
